# Segmentized Clear Channel Assessment for IEEE 802.15.4 Networks

**DOI:** 10.3390/s16060815

**Published:** 2016-06-03

**Authors:** Kyou Jung Son, Sung Hyeuck Hong, Seong-Pil Moon, Tae Gyu Chang, Hanjin Cho

**Affiliations:** 1 School of Electrical and Electronics Engineering, Chung-Ang University, Seoul 06974, Korea; skj9865@naver.com (K.J.S.); skadsc@gmail.com (S.H.H.); mczz01@dmc.cau.ac.kr (S.-P.M.); 2 Seoul SW-SoC R&BD Center, ETRI, Gyeong-gi 13480, Korea; hjcho@etri.re.kr

**Keywords:** clear channel assessment, carrier sense multiple access with collision avoidance (CSMA/CA), IEEE 802.15.4

## Abstract

This paper proposed segmentized clear channel assessment (CCA) which increases the performance of IEEE 802.15.4 networks by improving carrier sense multiple access with collision avoidance (CSMA/CA). Improving CSMA/CA is important because the low-power consumption feature and throughput performance of IEEE 802.15.4 are greatly affected by CSMA/CA behavior. To improve the performance of CSMA/CA, this paper focused on increasing the chance to transmit a packet by assessing precise channel status. The previous method used in CCA, which is employed by CSMA/CA, assesses the channel by measuring the energy level of the channel. However, this method shows limited channel assessing behavior, which comes from simple threshold dependent channel busy evaluation. The proposed method solves this limited channel decision problem by dividing CCA into two groups. Two groups of CCA compare their energy levels to get precise channel status. To evaluate the performance of the segmentized CCA method, a Markov chain model has been developed. The validation of analytic results is confirmed by comparing them with simulation results. Additionally, simulation results show the proposed method is improving a maximum 8.76% of throughput and decreasing a maximum 3.9% of the average number of CCAs per packet transmission than the IEEE 802.15.4 CCA method.

## 1. Introduction

IEEE 802.15.4 is uniquely designed to provide low-power consumption and low-cost for low-rate wireless personal area networks (LR-WPAN) [1,2,3]. Carrier sense multiple access with collision avoidance (CSMA/CA), which is employed by IEEE 802.15.4 medium access control (MAC) protocol, provides a low-power consumption feature to IEEE 802.15.4 [4]. Clear channel assessment (CCA) is an essential ingredient of CSMA/CA algorithms to sense the channel status [5]. Through CCA, a device can access the channel to transmit a packet or avoid a collision with other devices. In 802.15.4 based wireless devices, the portion of the power consumed by CCA is significant [6]. Therefore, it is very important that CCA algorithms provide not only throughput efficiency but also energy efficiency with a minimum number of CCA trials. Among the CCA modes, the energy detection based CCA mode consumes the least average power because its simplicity and response based passive operation [5].

The major drawback in the energy detection based CCA mode is its limited channel assessing behavior, which comes from simple threshold dependent channel busy evaluation. CSMA/CA performs two successive CCAs to determine the channel status. When CCA tries to check the channel status, it evaluates the energy level of the channel. The problem is that CCA assesses the channel as busy unconditionally if the energy level of the channel exceeds the threshold. Once CCA assesses the channel as busy, a device should wait for a random amount of time, which is called random backoff period, to perform the next CCA. This can lower the throughput performance and waste a lot of energy. However, even though the detected energy level exceeds the threshold, there is a chance to transmit a packet if CCA is performed at the end of a packet transmission. This is possible, because, after the end of a packet transmission, no other packet is transmitted through the channel. Through the precise channel assessing method, a device can catch that moment and transmit a packet.

Previous research for enhancing CSMA/CA algorithms by adjusting CCA can be divided into two categories. The first category involves updating the number of CCAs adaptively, as shown in [7,8], *etc.* The efficient backoff algorithm (EBA) updates the number of CCAs based on the probability of collision parameter [7]. The priority-based binary exponential backoff (PB-BEB) algorithm adjusts the number of CCAs by considering the fairness between devices [8]. These algorithms show better performance than the original CCA method in terms of fairness and throughput. However, varying the number of CCAs can cause more delay and energy consumption. The other category involves revising the CCA method without an updating process, as shown in [9,10,11], *etc.* Frame tailoring and short CCA (SCCA) algorithms perform CCA just one time by adjusting transmitted packets [9,10]. These algorithms lower the transmission delay and energy consumption, but do not increase the chance to transmit a packet. Additional carrier sensing (ACS) performs additional CCAs to increase the chance to transmit even if the CCAs assess the channel as busy [11]. However, additional CCAs could be a waste of time if a device cannot catch the chance of packet transmission through it.

This paper proposed and analyzed the segmentized CCA, which can increase the chance to transmit a packet without an updating process [12]. In contrast to the original CCA, the proposed CCA method treats the end of acknowledgment (ACK) packet as an idle state instead of as a busy state. Consequently, the proposed CCA method can improve throughput performance by saving one backoff period. The end of ACK packet is detected by measuring the energy distribution between the first half part and the second half part of the original CCA. The energy distribution between the two segmentized CCAs provides much more useful and effective information for the reliable detection of the end of ACK packet when compared with the total energy measured in the original CCA. Moreover, the specific energy distribution pattern of the segmentized CCA in the end of ACK packet situation (*i.e.*, the first half segmentized CCA is partially occupied by the ACK packet and the second half segmentized CCA must contain only noises) provides an effective means to design a reliable algorithm for the detection of the end of the ACK packet. To show the effectiveness of the proposed CCA method, the segmentized CCA is adapted to an IEEE 802.15.4 based network and compared with the ACS algorithm, which performs additional CCAs to increase the chance to transmit a packet.

The analytical model for IEEE 802.15.4 and the ACS algorithm have been developed in [11,13,14]. However, those papers do not reflect varying ACK packet transmission timing of IEEE 802.15.4. Since ACK packet transmission timing varies depending on the size of the data packet, analytic results are dependent on data packet size. Therefore, to obtain a precise analytical result, varying ACK packet transmission timing has to be considered. In this paper, the segmentized CCA is analyzed by considering all the possible network situations depending on the size of the data packet. Additionally, analytical results are compared with that of the ACS algorithm.

To confirm the validity of analytic results and the usability of the proposed algorithm, computer simulations have been performed. Through simulations, the accuracy of analytic results has been confirmed. Simulation results show the proposed method provides the maximum throughput gain of 8.76% and lowers the average number of CCAs per one packet transmission up to 3.9% better than the IEEE 802.15.4 CCA method.

## 2. Overview of IEEE 802.15.4

**Figure 1:**
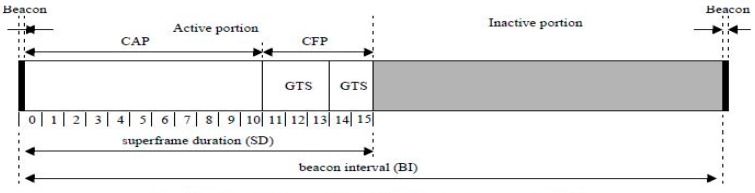
An example of the superframe structure.

In the slotted-mode of the IEEE 802.15.4 standard, the coordinator and its devices operate based on a superframe structure, which means the structure between each beacon frame. Figure 1 shows an example of the superframe structure [1]. The superframe is made up of an active and an inactive period. The active period contains 16 time slots, each consisting of 20 symbol periods, which are individually called unit backoff periods. In 2.4-GHz IEEE 802.15.4, each symbol contains 4 bits of data and the symbol rate is 62.5 (ksymbols/s), so the duration of the unit backoff period is 320 microseconds. The active period is divided into a contention access period (CAP) and a contention free period (CFP). In the CAP, data transmission is performed by using CSMA/CA and data transmission using allocated guaranteed time slot (GTS) is performed in the CFP.

When a device wants to transmit a data packet, the first thing the device does is wait for a random backoff period to avoid a collision with other devices. After the waiting time, the device performs two rounds of CCA successively to assess the channel status. If the energy level of the channel is above the threshold, the device conducts a random backoff process again. If the device decides the energy level of the channel is lower than the threshold during the two times of CCA, it transmits the data. To announce that a transmission was successful, a receiver transmits an ACK packet.

**Figure 2:**
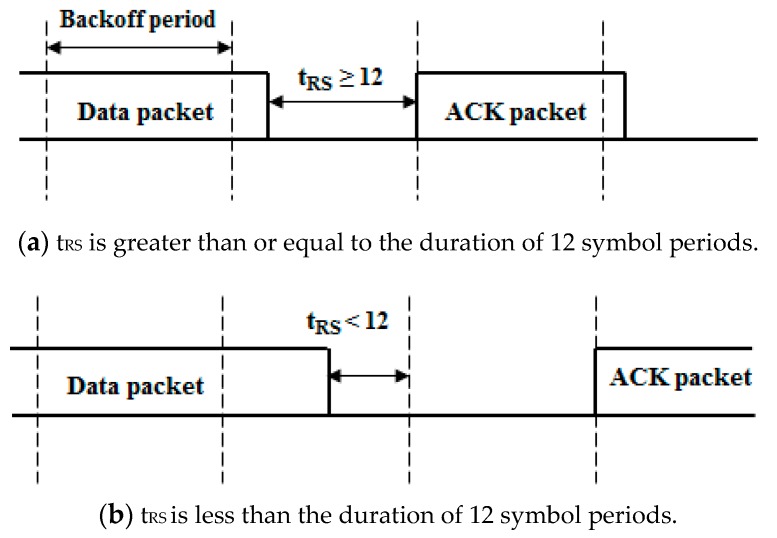
Acknowledgment (ACK) transmission timing depending on the reception of the last symbol of the data packet.

The ACK frame transmission timing depends on the reception of the last symbol of the data packet as shown in Figure 2. If the duration of the remaining symbol period t_RS_[sec] after the reception of the last symbol of the data packet is greater than or equal to the duration of 12 symbol periods, the ACK frame is transmitted at the next backoff period. If the remaining symbol is less than the duration of 12 symbol periods, the ACK frame is transmitted after a one backoff period delay.

## 3. Segmentized CCA

In this section, we introduce the segmentized CCA, which is the main idea for improving CSMA/CA discussed in this paper. The purpose of the segmentized CCA is to detect the end of an ACK packet transmission. Since no other packet is transmitted after the ACK packet transmission, a device can transmit a data packet if it detects the end of the ACK packet through its CCA. The segmentized CCA divides the original CCA into two parts to find the end of the ACK packet. By using the fixed size feature of the ACK packet, the segmentized CCA tries to detect the end of the ACK packet by comparing the energy level of two groups of CCA.

IEEE 802.15.4 CCA checks the channel energy during eight symbols of the backoff period and assesses the channel as busy if the energy level of the channel is above the threshold. Since the ACK packet is composed of 22 symbols, CCA assesses the channel as busy during two back off periods that are occupied by the ACK packet. However, the second backoff period of the ACK packet occupies just two symbols of the backoff period and no other packet is transmitted through the channel. Therefore, if CCA assesses the channel as idle at the second backoff period of the ACK packet, the device can transmit a data packet without collision.

The main idea of the segmentized CCA is to treat the end of ACK packet as an idle state instead of a busy state in the original CCA. The end of the ACK packet is detected by measuring the energy distribution between the first four symbols and the second four symbols of the original CCA. In the end of ACK packet situation, the first four symbol duration CCA detects two symbols of signal and the second four symbol duration CCA must contain only four symbols of noises. This provides a relatively large energy difference between the two segmentized CCAs, so the detection of the end of the ACK packet can be done reliably.

**Figure 3:**
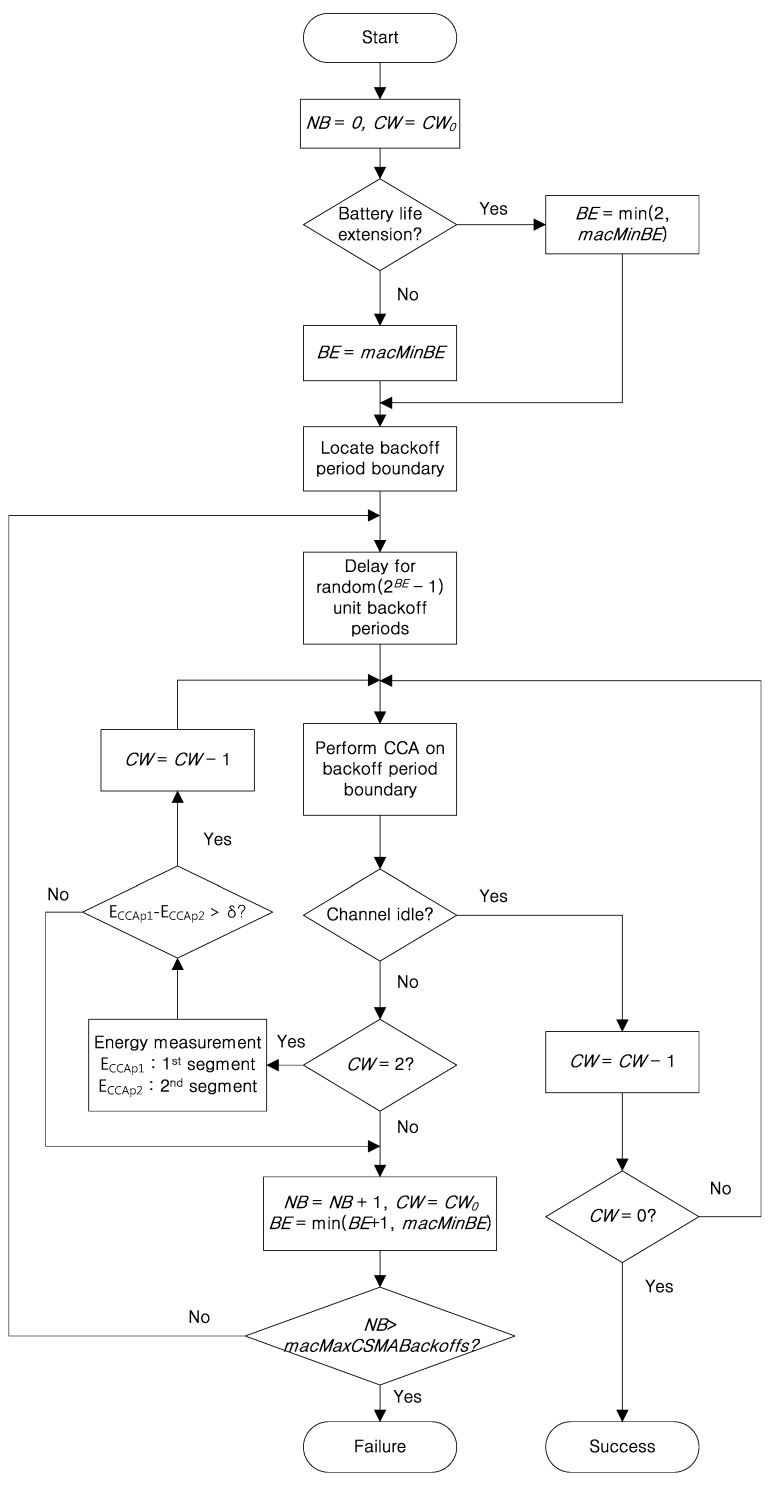
The flow chart of the segmentized clear channel assessment (CCA).

Figure 3 shows the flow chart of the segmentized CCA. If a device has a data packet to send, it waits for a random backoff period chosen between 0 and 2*^BE^* − 1. *BE* is the backoff exponent which is set to *macMinBE* as its initial value. The initial value of the *CW* (contention window) is set to 2, and it is decremented by 1 if the channel is assessed to be idle. When the value of *CW* comes to 0, the device decides data transmission is possible. After the random backoff period, the device performs a CCA that has a symbol length of eight. When the channel is assessed as busy, the proposed algorithm checks to see if it is the result of the first CCA by identifying *CW = 2*. If so, the device compares the energy level between the first half and second half of the CCA (E_CCAp1_, E_CCAp2_). E_CCAp1_ and E_CCAp2_ represent the energy of the first half and the second half symbols of the original CCA, respectively. The values of E_CCAp1_ and E_CCAp2_ depend on how long a data or an ACK packet occupies the CCA period. In the end of the ACK packet situation, E_CCAp1_ shows the energy of the signal during the two (2) symbol period and E_CCAp2_ denotes the energy of noise during the four (4) symbol period. If E_CCAp1_ − E_CCAp2_ > δ, the device performs a second CCA since it decides the CCA was performed at the end of a packet transmission. Otherwise, the device performs a random backoff. The threshold δ decides whether the busy channel is caused by the end of a packet transmission or not. The number of CCA failures allowed is up to *macMaxCSMABackoffs*.

**Figure 4:**
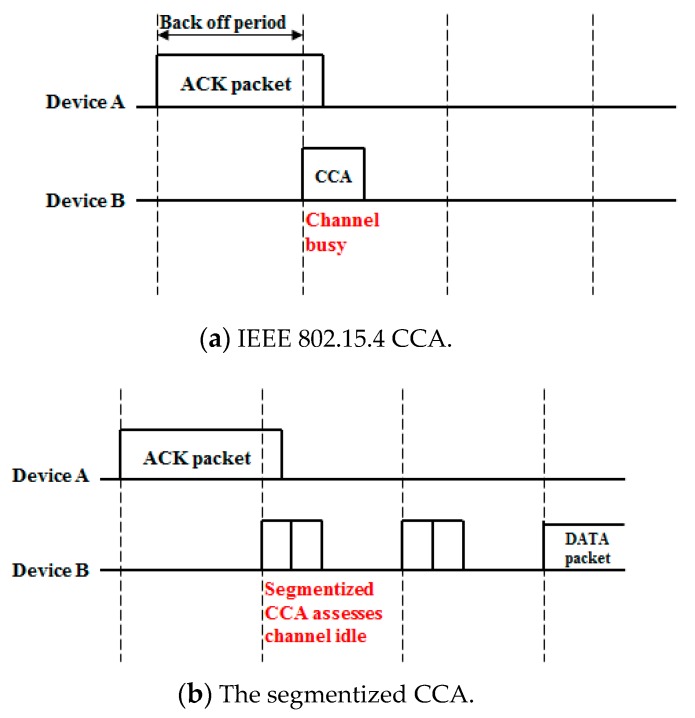
CCA performed at the end of an ACK transmission.

**Figure 5:**
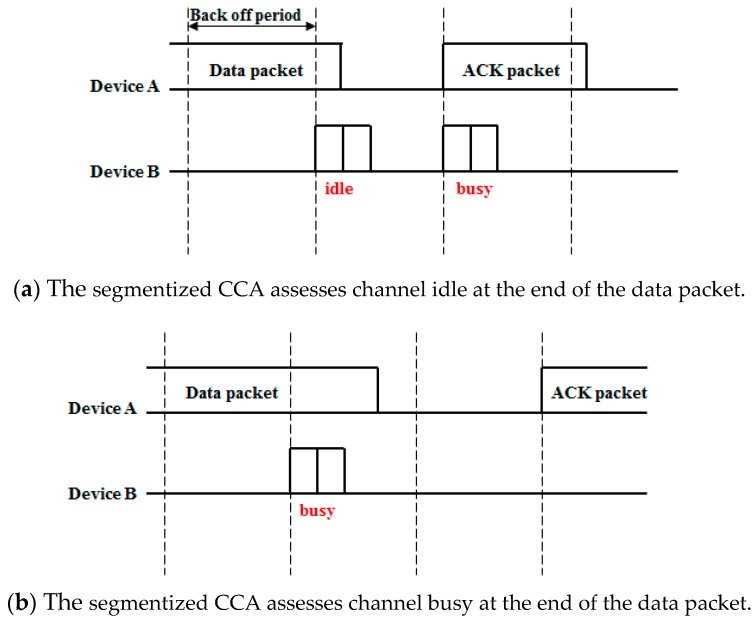
The segmentized CCA performed at the end of a data transmission.

Figure 4 shows the difference between the IEEE 802.15.4 CCA and the proposed CCA. As shown in Figure 4a, the IEEE 802.15.4 CCA assesses the channel as busy at the end of the ACK transmission so it performs a random backoff period. On the other hand, after the segmentized CCA has been done, a data packet is transmitted because the segmentized CCA assesses the channel as idle at the end of the ACK transmission. When the segmentized CCA is conducted at the end of a data transmission, the result depends on the last backoff period symbol size that was occupied by a data packet. Examples of these situations are shown in Figure 5.

## 4. Analysis of the Segmentized CCA and Comparison with the ACS Algorithm

In this section, the segmentized CCA is mathematically analyzed and compared with the ACS algorithm. Both the segmentized CCA and the ACS algorithms try to increase the chance of packet transmission by detecting an ACK packet. It is natural curiosity to strive towards finding a better algorithm if there are many possible algorithms that can be used to solve the same problem. To resolve this, the authors analyzed the proposed algorithm and compared throughput performance between the two algorithms. To get more useful results, various packet sizes are considered, as this factor affects the transmission timing of an ACK packet.

### 4.1. Analysis of the Segmentized CCA

**Figure 6:**
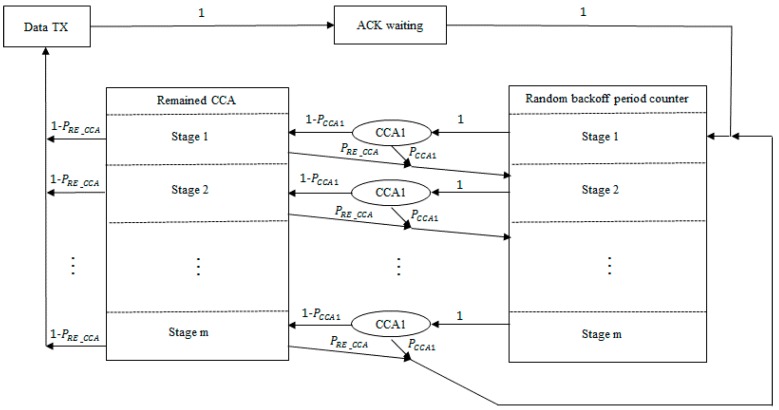
Markov model for the segmentized CCA algorithm and the additional carrier sensing (ACS) algorithm under saturated traffic condition.

In this subsection, the proposed algorithm was mathematically analyzed using the case of acknowledged uplink data transmission with saturated traffic conditions. Various packet sizes were considered to reflect different ACK packet transmission timing depending on the packet size. Figure 6 shows a Markov model representing the behavior of a single device. The Markov model can be applied to both the segmentized CCA and the ACS algorithms by varying *P_RE_CCA,_* which represents the probability of assessing the channel as busy except for the first CCA.

P_CCA1_ is the probability of assessing the channel as busy during the first CCA. P_CCA1_ directly affects throughput performance because it is one of the major parameters for probability of successful channel accessing. The first CCA is performed after the random backoff period counter expires. When the first CCA assesses the channel as idle, the next CCA is performed. Failure of the CCA causes the next random backoff if the number of random backoff trials has not exceeded *macMaxCSMABackoffs*. Let ∅ be the probability that the device attempts its first CCA [14]. P_CCA1_ can be expressed by Equation (1) [13,14].

(1) $$P_{CCA1}=L^{*}\left[1-{\left(1-\emptyset \right)}^{N-1}\right]\left(1-P_{CCA1}\right)\left(1-P_{RE\_CCA}\right)$$

where $L^{*}=L_{Data}+L_{ACK}\times \left(1-P_{netcol}\right)$ and *N* is the number of devices. $L_{Data}$, $L_{ACK}$ denote the data and ACK packet transmission duration measured in backoff periods, respectively. $P_{netcol}$ is the probability [14] that a collision is seen on the channel on the condition that a transmission was going on and is given by

(2) $$P_{netcol}=1-\frac{N\emptyset {\left(1-\emptyset \right)}^{N-1}}{1-{\left(1-\emptyset \right)}^{N}}$$

$L^{*}$ and $P_{RE\_CCA}$, which are included in the formula of $P_{CCA1}$, depend on the network situation. An existing empty backoff period between the data and ACK packets makes a difference. Because of this feature, respective different network situations have to be considered when $L^{*}$ and $P_{RE\_CCA}$ are analyzed. For the segmentized CCA, CCAs are performed only two times so $P_{RE\_CCA}$ can be considered as $P_{CCA2},$ which means the probability of assessing the channel as busy during the second CCA.

**Figure 7:**
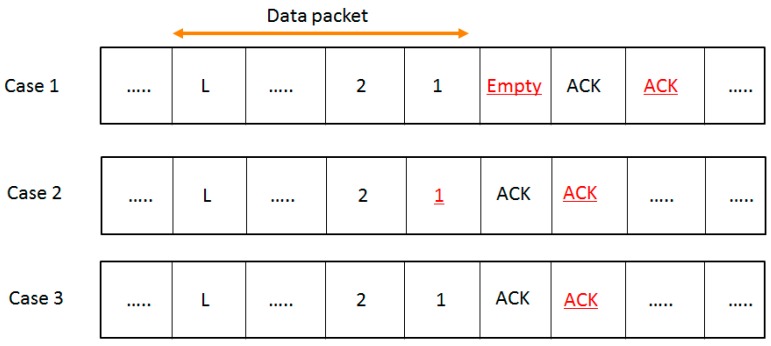
Network situations depending on variable packet sizes when the segmentized CCA is used.

Figure 7 shows possible network situations when the segmentized CCA is used. The underline indicates that the segmentized CCA assesses the channel as idle at that moment. Analyses have been done in the following cases.

Case 1: An empty backoff period exists between the data and ACK packets. In this case, the proposed algorithm senses the channel as idle at the end of the ACK transmission. Therefore, $L^{*}$ can be considered as

(3) $$L^{*}=L_{Data}+\left(L_{ACK}-1\right)\times \left(1-P_{netcol}\right)$$

Since the segmentized CCA is used, only two rounds of CCA are performed. Therefore, the meaning of $P_{RE\_CCA}$ is the probability of assessing the channel as busy during the second CCA. In [14], the probability to sense the channel as busy on the second CCA is defined as the following.

(4) $$P_{RE\_CCA}=\left(channel\;busy\;caused\;by\;data\;packet\right)+\left(channel\;busy\;caused\;by\;ACK\;packet\right)=\left[1-\frac{\left(1-P_{netcol}\right)+1}{\left(2-P_{netcol}+\frac{1}{1-{\left(1-\emptyset \right)}^{N}}\right)}\right]\times \left(1-{\left(1-\emptyset \right)}^{N-1}\right)+\frac{1-P_{netcol}}{\left(2-P_{netcol}+\frac{1}{1-{\left(1-\emptyset \right)}^{N}}\right)}$$

for a large *N*, it can be simplified to

(5) $$P_{RE\_CCA}=\frac{2-P_{netcol}}{\left(2-P_{netcol}+\frac{1}{1-{\left(1-\emptyset \right)}^{N}}\right)}$$

Case 2: An empty backoff period does not exist and the last backoff period occupied by the data packet is under eight symbols. In this case, the proposed algorithm senses the channel as idle both at the end of the data and the ACK transmission. $L^{*}$ can be considered as

(6) $$L^{*}=\left(L_{Data}-1\right)+\left(L_{ACK}-1\right)\times \left(1-P_{netcol}\right)$$

$P_{RE\_CCA}$ is the same with that of Case 1 because the segmentized CCA senses the channel as idle at the end of the data transmission.

$$P_{RE\_CCA}=\frac{2-P_{netcol}}{\left(2-P_{netcol}+\frac{1}{1-{\left(1-\emptyset \right)}^{N}}\right)}$$

Case 3: An empty backoff period does not exist and the last backoff period occupied by the data transmission is eight (8) symbols. In this case, the segmentized CCA senses the channel as idle at the end of the ACK transmission. $L^{*}$ is the same with that of Case 1 so

$$L^{*}=L_{Data}+\left(L_{ACK}-1\right)\times \left(1-P_{netcol}\right)$$

$P_{RE\_CCA}$ is different with the above cases because second CCA busy is caused by an ACK packet.

(7) $$P_{RE\_CCA}=\left(channel\;busy\;caused\;by\;data\;packet\right)=\left[1-\frac{\left(1-P_{netcol}\right)+1}{\left(2-P_{netcol}+\frac{1}{1-{\left(1-\emptyset \right)}^{N}}\right)}\right]\times \left(1-{\left(1-\emptyset \right)}^{N-1}\right)=\frac{\frac{1}{1-{\left(1-\emptyset \right)}^{N}}}{2-P_{netcol}+\frac{1}{1-{\left(1-\emptyset \right)}^{N}}}\times \left(1-{\left(1-\emptyset \right)}^{N-1}\right)$$

for a large *N*, it can be simplified to

(8) $$P_{RE\_CCA}=\frac{1}{2-P_{netcol}+\frac{1}{1-{\left(1-\emptyset \right)}^{N}}}\;$$

### 4.2. Throughput Comparison between the Segmentized CCA and the ACS Algorithm

In this subsection, a throughput comparison between the segmentized CCA and the ACS algorithms has been performed. Before doing that, additional analyzing of the ACS algorithm is performed since the analysis of the ACS algorithm in [11] considered only one network situation. With all the formulas considering all the network situations, throughput comparison has been done for each packet size case.

The ACS algorithm performs three rounds of CCA, hence $P_{RE\_CCA}$ can be defined as

(9) $$P_{RE\_CCA}=\;P_{CCA2}\times P_{CCA3}$$

where $P_{CCA2}$, $P_{CCA3}$ are the probability of assessing the channel as busy during the second and third CCA, respectively. In [11], the ACS algorithm has been analyzed considering an empty backoff period between the data and ACK packets. The formulas of $P_{CCA2}$, $P_{CCA3}$ are

(10) $$P_{CCA2}=\frac{3-2P_{netcol}}{3-2P_{netcol}+\frac{1}{1-{\left(1-\emptyset_{ACS}\right)}^{N}}}$$

(11) $$P_{CCA3}=\frac{2-P_{netcol}}{3-2P_{netcol}+\frac{1}{1-{\left(1-\emptyset_{ACS}\right)}^{N}}}$$

where $\emptyset_{ACS}$ is the probability that a device performs its first CCA when the ACS algorithm is used [11]. $P_{CCA1}$ is the same with the segmentized CCA, with the exception that ∅ is changed into $\emptyset_{ACS}$.

However, $P_{CCA2}$, $P_{CCA3}$ are different from Equations (10) and (11) if there is no empty backoff period between the data and ACK packets. In this case, the third CCA cannot assess the channel as idle. This means the data transmission caused by the third CCA no longer happens. Therefore, if the second CCA assesses the channel as busy then the third CCA always assesses the channel as busy, so $P_{CCA3}=1$ and $P_{CCA2}$ is the same as Equation (8) except for ∅.

**Figure 8:**
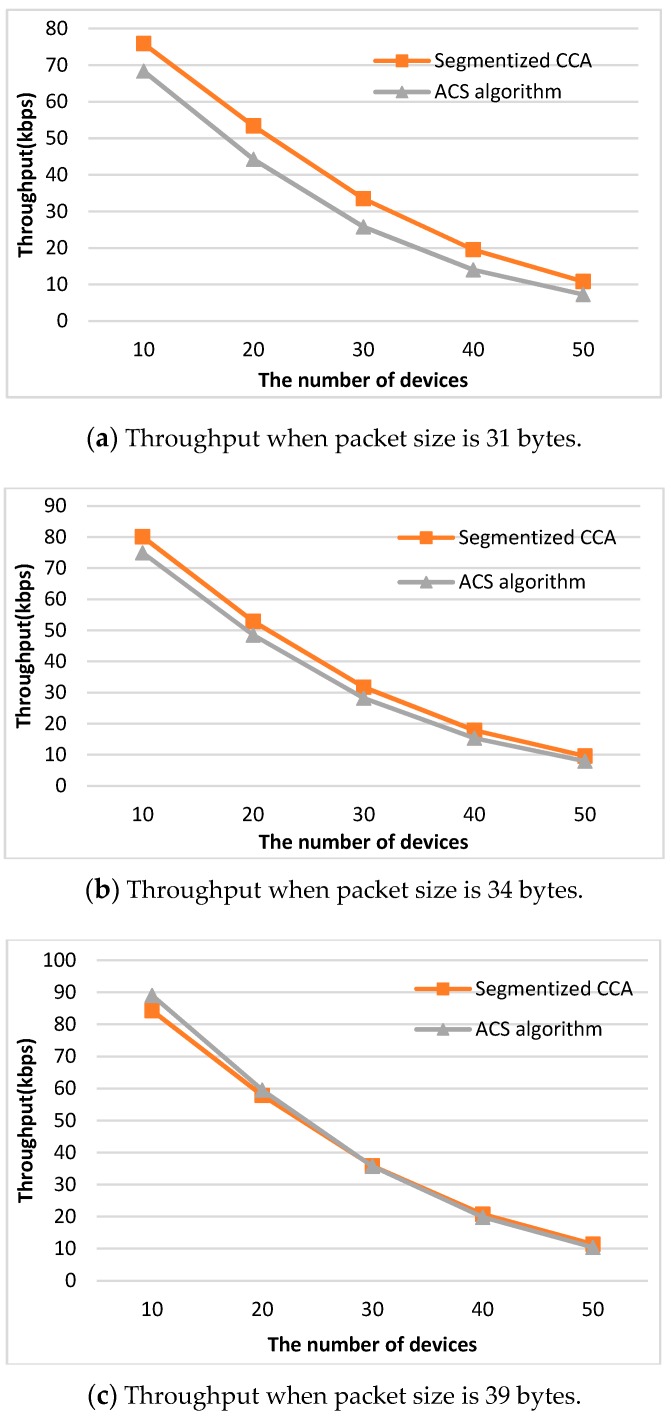
Comparison of analysis of throughput values between the segmentized CCA and the ACS algorithm.

A throughput formula is given by

(12) $$S=L_{Data}N\emptyset {\left(1-\emptyset \right)}^{N-1}\left(1-P_{CCA1}\right)\left(1-P_{RE\_CCA}\right)\times A=AL_{Data}P_{success}$$

where $A=\frac{80bit}{0.32ms}$ is a normalization constant to convert to bps when the bit rate is 250 kbps [14]. To reflect all the possible network situations depending on the packet size, three types of packet sizes (31, 34, and 39 bytes) are used. Packet sizes of 31, 34, and 39 bytes represent Case 2, Case 3, and Case 1 of Figure 7, respectively. Figure 8 shows a comparison of analysis of the throughput values between the segmentized CCA and the ACS algorithms.

**Figure 9:**
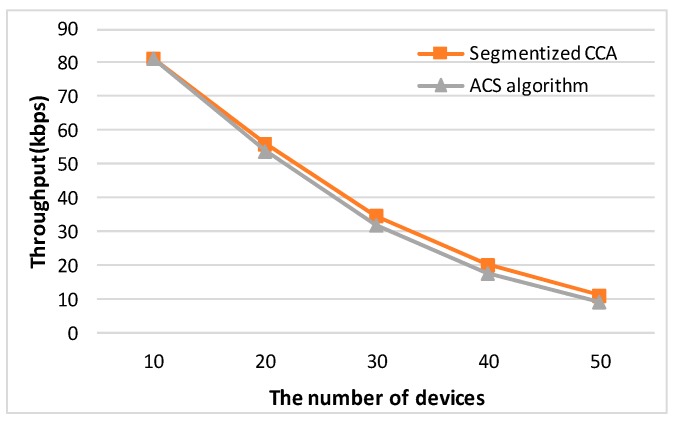
Comparison of analysis throughput values between the segmentized CCA and the ACS algorithms when three packet sizes are used at the same time.

When the packet sizes are 31 and 34 bytes, which do not make an empty backoff period between the data and ACK packets, the proposed CCA method outperforms the ACS algorithm. The reason is that the ACS algorithm only works when an empty backoff period exists. When the packet size is 39 bytes and the number of devices is 10 and 20, the ACS algorithm shows better performance. However, in the real world, transmitted packet sizes are random, so all three cases happen simultaneously. Figure 9 shows a comparison of analysis of the throughput values between the segmentized CCA and the ACS algorithms when three packet sizes are used at the same time.

## 5. Simulation

Simulation experiments were performed to validate the proposed analytical results and to verify the effectiveness of the proposed method by using the Visual C++ program. In the simulation, a star topology with one coordinator and a lot of devices are considered, and the uplink saturated traffic condition is assumed. Six types of packet sizes (31, 34, 39, 51, 54, and 59 bytes) were used to reflect all the possible network situations that are discussed in the analysis section. When the three types of packets (*i.e.*, 31, 34 and 39 bytes) are used at the same time, they are uniformly distributed so the proportion is 20%, 20%, and 60%, respectively. For the other three packets (*i.e.*, 51, 54, and 59 bytes), the same proportions of 20%, 20%, and 60%, respectively, are used in the simulation. Table 1 shows the system parameters used for the simulation.

### 5.1. Analysis Validation of the Segmentized CCA and the ACS Algorithm

**Figure 10:**
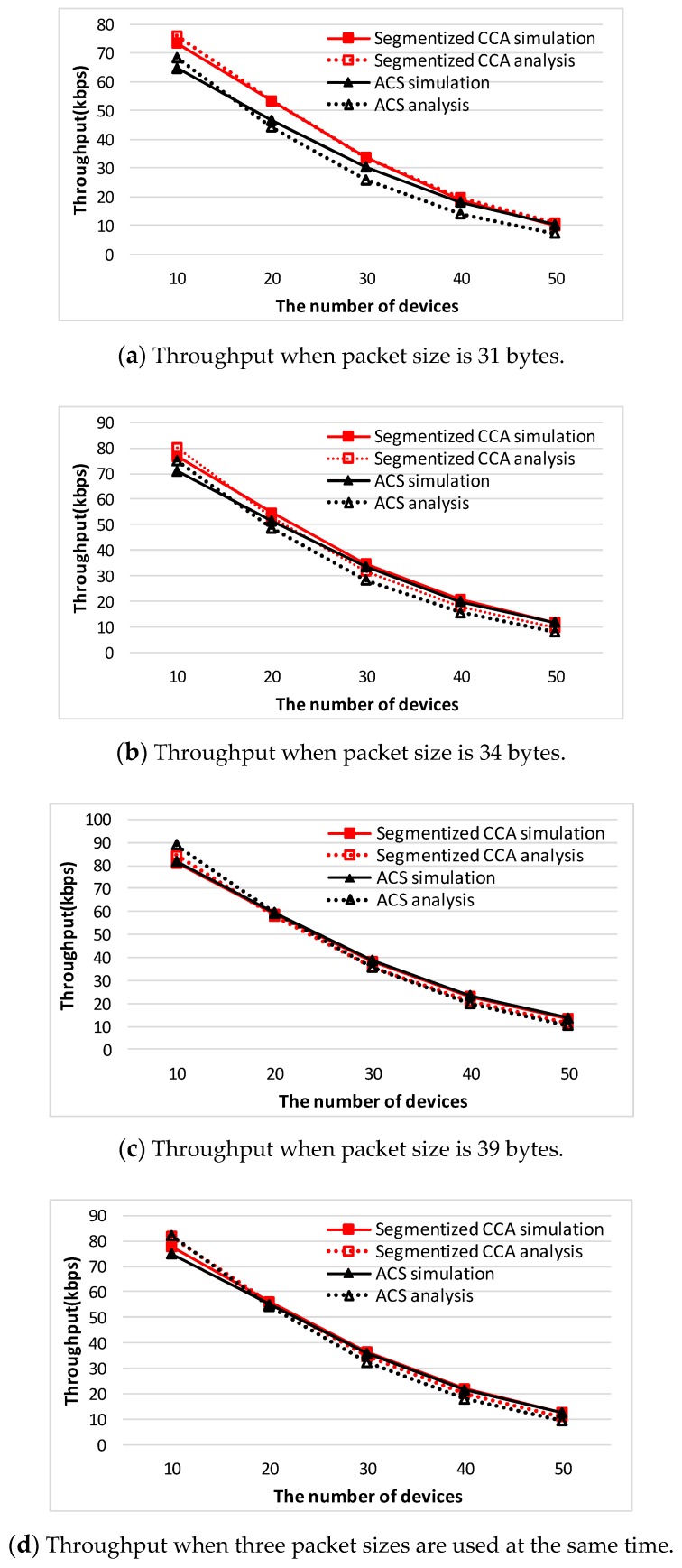
Throughput comparison between the segmentized CCA and the ACS algorithm for the packet size pattern of 31, 34, and 39 bytes.

**Figure 11:**
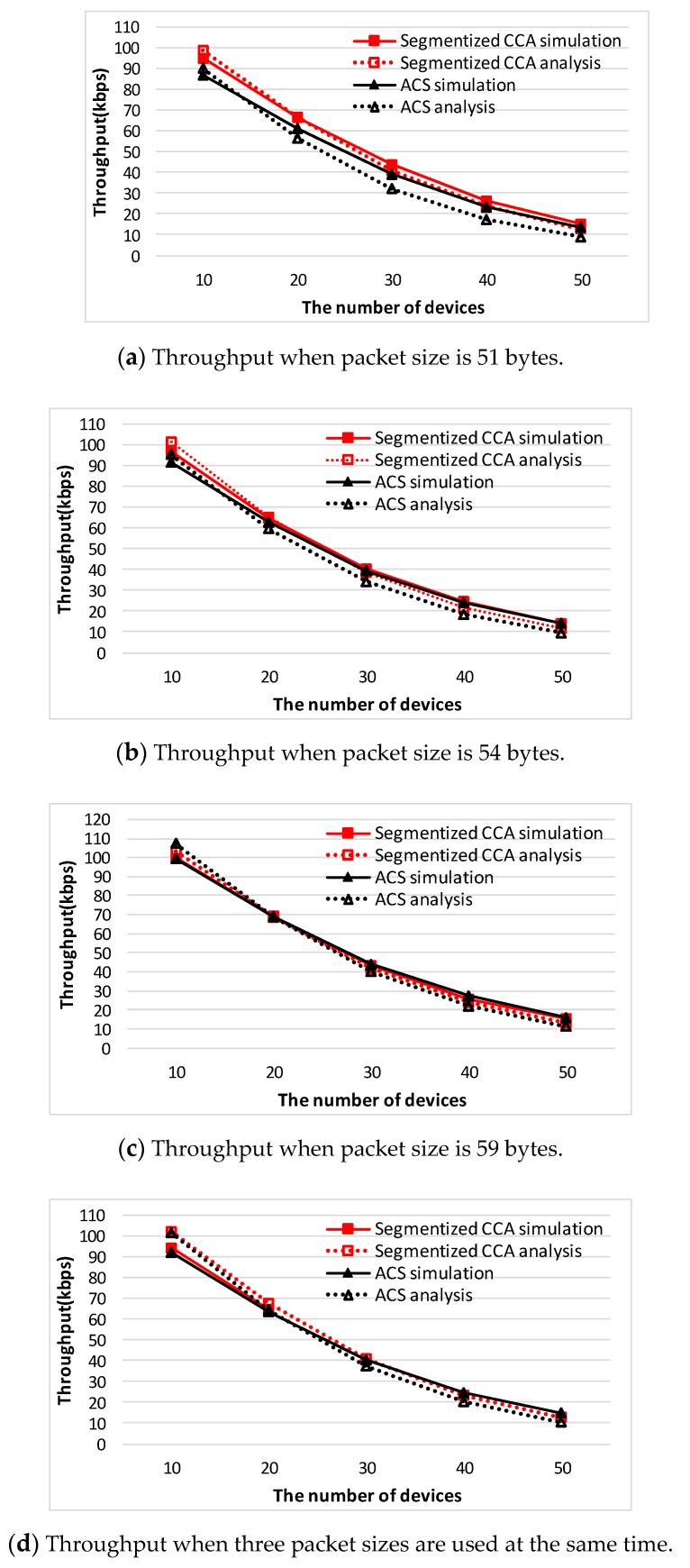
Throughput comparison between the segmentized CCA and the ACS algorithm for the packet size pattern of 51, 54, and 59 bytes.

In the analysis section, the segmentized CCA and the ACS algorithms are analyzed for three different network situations. To confirm those results are valid, comparison with the simulation results has been done. Figure 10 and Figure 11 show throughput comparison between the analysis and the simulation result of both CCA algorithms. It is observed that there exists a slight mismatch between the analysis and the simulation result. The mismatch is the result of approximations in accordance with the variations of the number of device used in the derivation of the throughput performance.

### 5.2. Performance Comparison among CCA Algorithms

**Figure 12:**
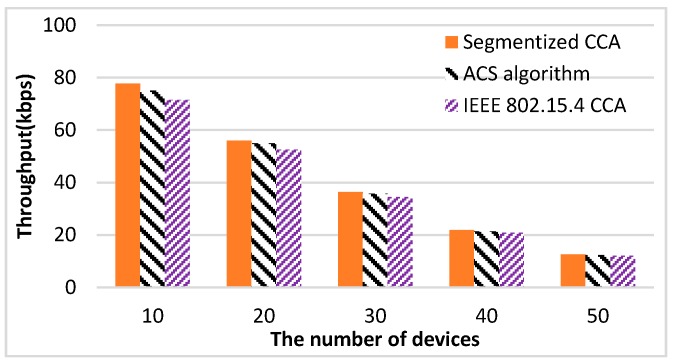
Throughput results of three CCA methods.

**Figure 13:**
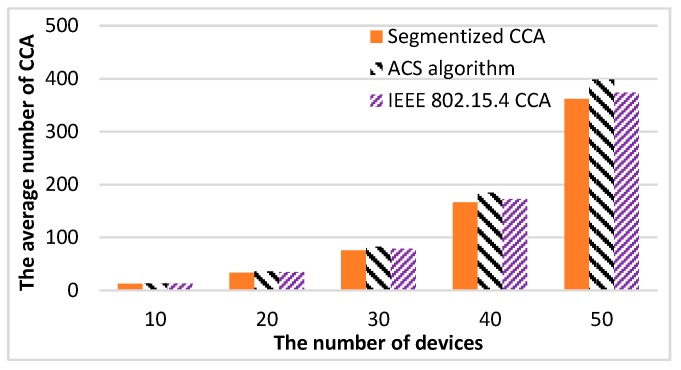
The average number of CCAs for three CCA methods.

The performance of the proposed algorithm is compared with the IEEE 802.15.4 CCA and ACS algorithms. The performance of the throughput and the average number of CCAs show similar results for the two sets of packet size patterns. For the detailed illustration of the comparison results, the throughput and the average number of CCA for the packet size pattern of 31, 34, and 39 are shown in the Figure 12 and Figure 13 and Table 2 and Table 3. Figure 12 shows the results of the throughput performance measurements *versus* the number of devices. The proposed method shows the best throughput performance regardless of the number of devices. Since the ACS algorithm focuses on the failure of the second CCA caused by an ACK packet, the ACS algorithm does not work if there is no empty backoff period between a data packet and the ACK packet. On the other hand, the segmentized CCA works all the time since the proposed method focuses on detecting the end of the packet transmission at the first CCA. Table 2 shows the throughput increasing rate comparing the ACS algorithm and the segmentized algorithms with the IEEE 802.15.4 CCA. The proposed method increases the throughput performance up to 8.76% compared with the IEEE 802.15.4 CCA method. Since the collision effect increases as the number of devices increases, the difference of throughput among the three CCA methods gets smaller.

Figure 13 shows the results of the average number of CCA (total number of CCA/total number of successful packet transmissions) measurements *versus* the number of devices. Performing CCA requires a large amount of energy, as much as transmitting or receiving a packet [6]. This means lowering the number of average CCAs can increase the energy efficiency of CSMA/CA. The proposed method shows the lowest average number of CCAs at all times. The ACS algorithm shows the highest average number of CCAs because the ACS algorithm always performs CCA three times if the second CCA assesses the channel as busy. Table 3 shows the rate of the average number of CCAs increasing comparing the ACS algorithm and the segmentized algorithms with IEEE 802.15.4 CCA. The proposed method lowers the average number of CCAs up to 3.9% compared with the IEEE 802.15.4 CCA method. From these results, it can be confirmed that the proposed method provides the highest energy efficiency over other CCA algorithms.

## 6. Conclusions

In this paper, the segmentized CCA for IEEE 802.15.4 networks has been proposed. The proposed method solves the unnecessary busy channel evaluation problem that comes from the simple threshold dependent channel assessment of the previous method. The proposed method divides IEEE 802.15.4 CCA into two parts to assess whether the busy channel is caused by the end of packet transmission. If the first CCA is performed at the end of the ACK packet, it assesses the channel as idle and the device transmits a data packet after performing a second CCA.

The proposed method has been analyzed in the case of acknowledged uplink data transmission with saturated traffic conditions. Various packet sizes were also considered to reflect different ACK packet transmission timing. Through the simulation, the validation of analytical results has been confirmed. Also, throughput comparison among the segmentized CCA, the ACS algorithm, and the IEEE 802.15.4 CCA has been shown. The proposed method provides the maximum throughput gain of 8.76% better than the IEEE 802.15.4 CCA method. In addition, the proposed method lowers the average number of CCAs per one packet transmission up to 3.9% better than the IEEE 802.15.4 CCA method.

## Figures and Tables

**Table 1:** The system parameters used in the simulation.

Parameter	Value
Channel bandwidth	250 kbps
*CW*	2
*aMinBE*	3
*aMaxBE*	5
*macMaxCSMABackoffs*	5
Packet size	31, 34, 39 bytes 51, 54, 59 bytes

**Table 2:** Throughput increasing rate in comparison with the IEEE 802.15.4 CCA method.

The Number of Devices	CCA Method	
	ACS Algorithm	Segmentized CCA
10	4.88%	8.76%
20	4.69%	6.74%
30	3.86%	5.79%
40	2.44%	4.85%
50	2.56%	4.09%

**Table 3:** The average CCA increasing rate compared to the IEEE 802.15.4 CCA method.

The Number of Devices	CCA Method	
	ACS Algorithm	Segmentized CCA
10	3.13%	−3.9%
20	4.08%	−3.5%
30	5.43%	−3.52%
40	6.81%	−3.7%
50	6.63%	−3.26%
